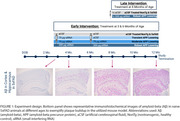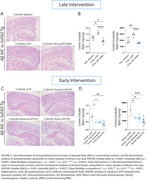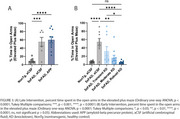# Small Interfering RNA Targeting Amyloid‐Beta Precursor Protein Reduces Alzheimer’s Disease Pathology in 5xFAD Mice

**DOI:** 10.1002/alz70859_098351

**Published:** 2025-12-25

**Authors:** Dominique Taillie, Diana Cha, Gina Ferraro, Kelsey Bittner, Alexandre Sostelly, Timothy Mooney, Kirk Brown

**Affiliations:** ^1^ Alnylam Pharmaceuticals, Cambridge, MA USA

## Abstract

**Background:**

Amyloid‐beta precursor protein (APP) is the source of all amyloid‐beta (Aβ), the amyloidogenic peptide that aggregates in Alzheimer’s disease (AD). Lowering APP mRNA expression with RNA interference (RNAi) is a novel treatment strategy under investigation for AD. The 5xFAD mouse model of AD overexpresses APP and PSEN1 with five familial AD mutations, resulting in robust pathology, including amyloid plaques, microgliosis, inflammation, synaptic loss, neuronal loss, and behavioral changes. This work evaluates the effects of early and late intervention with an APP‐lowering small interfering RNA (siRNA) in this established AD model.

**Method:**

APP‐targeting siRNAor artificial CSF (aCSF; control) was administered by intracerebroventricular injection in 5xFAD mice at late (8‐months‐old) and early (12‐weeks‐old) disease stages, and in age‐matched wild‐type mice. Late intervention mice received either aCSF or 300µg single dose. Early intervention mice received one of four dose regimens: control (aCSF:aCSF), transient lowering (75µg APP siRNA:aCSF), moderate sustained lowering (75µg APP siRNA:75µg APP siRNA), or robust sustained lowering (300µg APP siRNA:300µg APP siRNA). The impact of late and early siRNA intervention on molecular, biochemical, histological, and disease‐related behavioral outcomes (assessed using the elevated plus‐maze and open‐field assay) were assessed in 12‐month‐old mice (Figure 1).

**Result:**

Treatment in the late intervention group lowered amyloid burden to levels below the 8‐month baseline (Figure 2A, 2B). Compared with age‐matched aCSF mice, late treatment decreased pathogenic amyloid species, Aβ40 and Aβ42, in CSF and tissue, reduced markers of glial inflammation, and reduced plasma neurofilament light chain (NfL). No significant behavioral changes were noted (Figure 3A).

Treatment in early intervention groups lowered amyloid burden in tissue (Figure 2C, 2D), reduced markers of glial inflammation, reduced plasma NfL, and decreased anxiety‐like behavior, all in a dose‐dependent manner. Notably, robust, sustained lowering of APP completely prevented the emergence of disease‐associated anxiety‐like behavior (Figure 3B).

**Conclusion:**

Treatment with APP‐lowering siRNA reduced AD pathology in the 5xFAD mouse model. Early intervention with the highest dose regimen prevented the emergence of behavioral deficits and substantially improved many aspects of disease phenotype. These results support the continued development of mivelsiran, an investigational, first‐in‐class APP‐lowering RNAi therapeutic, in patients with AD (NCT05231785).